# Association between Childhood Maltreatment History and Premenstrual Syndrome

**DOI:** 10.3390/ijerph18020781

**Published:** 2021-01-18

**Authors:** Kanako Ito, Satomi Doi, Aya Isumi, Takeo Fujiwara

**Affiliations:** 1 Cocokara Women’s Clinic, Nagoya 461-0001, Japan; itohlth@tmd.ac.jp; 2 Department of Global Health Promotion, Tokyo Medical and Dental University (TMDU), Tokyo 113-8519, Japan; doi.hlth@tmd.ac.jp (S.D.); isumi.hlth@tmd.ac.jp (A.I.); 3 Japan Society for the Promotion of Science, Tokyo 102-0083, Japan

**Keywords:** premenstrual syndrome, adverse childhood experience, child maltreatment, abuse

## Abstract

Childhood maltreatment history has known relationships with various mental and physical diseases; however, little is known about its association with premenstrual syndrome (PMS). In this study, we investigated the association between childhood maltreatment history and PMS among young women in Japan. In a Japanese city, we approached 3815 women aged 10–60 years who visited a gynecology clinic and one general practice clinic. A questionnaire on childhood maltreatment history and PMS was administered to them. We observed that women with histories of childhood maltreatment demonstrated a significantly increased risk of PMS compared with those without such histories (odds ratio: 1.47, 95% confidence interval: 1.20–1.81). Particularly, women with childhood physical or emotional abuse demonstrated a stronger association with PMS, whereas other forms of childhood maltreatment (emotional neglect, witnessing of intimate-partner violence, or sexual abuse) were not associated with PMS. Our results illustrate that childhood maltreatment may be a risk factor for PMS.

## 1. Introduction

Mental and physical symptoms of premenstrual syndrome (PMS) have a significant impact on women, incurring a substantial public health burden [1]. PMS is a complex disorder characterized by mood changes such as irritability, depression, and physical symptoms limited to 7–14 days before the onset of the late luteal phase of the menstrual cycle [2]. Some authors suggest that PMS is not a fixed unitary syndrome but rather a diagnostic label that is socially applied to negative premenstrual changes, which may be specific to westernized cultures [3]. Thus, epidemiological surveys are needed to capture the prevalence of PMS in different contexts. In Japan, approximately 75–95% of women are reported to experience PMS during their reproductive life [4,5]. Furthermore, approximately 5% of women are known to experience severe symptoms of PMS, which affect their personal and social relationships or work [6]. In the United States, the annual cost per woman who suffers from PMS was estimated to be USD 5000 [7]. However, these figures are underestimated; this is because a large proportion of cases go undiagnosed due to the difficulties encountered by clinicians during diagnosis and the tendency of many women to not consult a doctor for PMS-related symptoms [4,8].

The known risk factors for PMS are hormonal changes, stress, serotonergic dysfunction, and unhealthy behaviors, such as lack of exercise, short sleep duration, or poor diet [9]. However, we chose to investigate childhood maltreatment as a risk factor of PMS because childhood maltreatment histories are known to be related to various mental and physical diseases [10,11,12]. For example, Felitti et al. reported that people who had experienced at least four different types of childhood maltreatment had a 4- to 12-fold increase in health risks for alcoholism, drug abuse, depression, and suicide, compared with those who had experienced no such episodes [11]. Several studies have investigated the association between childhood maltreatment history (emotional, physical, and sexual abuse) and PMS in different races [13]. For example, using the Nurses’ Health Study in the United States, women reporting the highest level of emotional abuse were found to have 2.6-times the risk of PMS compared with those reporting none [14]. Although this finding is robust, it may not be generalizable to all women because nurses may remember their childhood maltreatment history or express PMS symptoms differently than women who are not trained in clinical settings.

To date, most studies conducted on this topic have been limited to either case reports or those with relatively small sample sizes; hence, we decided to investigate a larger cohort. Beyond this, we felt it was important to extend the study of the association between childhood maltreatment history and PMS to other cultures, such as Japan, where the prevalence of domestic violence, abuse, and sexual abuse is lower than that in the United States [15].

Thus, the present study investigates the association between childhood maltreatment and PMS among women in a clinical setting in Japan.

## 2. Materials and Methods

### 2.1. Participants

This study is a case–control study. In Nagoya City in Aichi Prefecture, Japan, we targeted all female patients (aged 10–60 years) who visited Cocokara Women’s Clinic (gynecology) and Cocokara Heart Clinic (psychiatry and general internal medicine) between April 2016 and May 2018 (a total of 3940 women). Upon their first visit to each clinic, an additional questionnaire was administered to the target patients along with the questionnaire on demographic characteristics. Patients sought consultation not only for the treatment of symptoms, such as menstrual cramps and irregular menstruation, but also for other reasons, including cervical cancer screening, breast cancer screening, and vaccination. Thus, we were able to administer the questionnaire to individuals without gynecological diseases.

We collected the responses of 3815 women but excluded those with missing information concerning the presence or absence of PMS symptoms (including those without information on menses) and those that had missing information in all questions relating to childhood maltreatment history.

### 2.2. Measurements

#### 2.2.1. Assessment of PMS

According to the Japanese Society of Obstetrics and Gynecology, PMS is defined as a “mental or physical symptom lasting from 3 to 10 days before menstruation, which diminishes or disappears with menstruation” [16]. Although the definition and onset frequency of PMS differ between Japan and the United States, the American College of Obstetrics and Gynecology (ACOG) PMS diagnostic criteria are often utilized in Japan [17]. Thus, using the ACOG definition, we asked the following 12 questions concerning the diagnosis of PMS: “Of the following symptoms, select all that lasted for 3 to 13 days before menstruation in the previous 3 menstrual cycles: (1) depressed mood (feeling of depression), (2) increased anger, (3) frustration, (4) anxiety, (5) confusion, (6) withdrawal from housework and work (taking time off from school or work), (7) breast tightness, (8) abdominal fullness (abdominal pain), (9) headache, (10) edema, (11) lower-back pain (joint or muscle pain), and (12) weight gain.” Women who responded affirmatively to at least one question were indicated as having PMS.

#### 2.2.2. Childhood Maltreatment History

To assess childhood maltreatment history, five questions were asked with responses given on a 4-point Likert scale: 4 = “strongly agree,” 3 = “somewhat agree,” 2 = “somewhat disagree,” and 1 = “strongly disagree.” We defined childhood maltreatment as responses of 3 or 4. The following questions were asked: “My parent/s did not take care of me,” “There was intimate-partner violence (IPV) at home,” “I was physically abused, such as hitting or kicking by my parent/s,” “I was psychologically abused, such as being told terrible things by my parent/s,” and “I experienced any type of sexual trauma from an acquaintance when I was a child.” The questions were developed based on previous studies of adverse childhood experiences in Japan [11,18,19,20,21].

#### 2.2.3. Covariates

The questionnaire also included questions about marital status, living with a child or children, smoking, alcohol consumption, economic hardship, and domestic violence. Marital status was determined by the following question: “Please state your marriage history.” Respondents answered using “1 = unmarried,” “2 = married or common-low marriage,” “3 = divorce,” “4 = bereavement,” or “5 = others.” Respondents were categorized into the following three groups: “never married,” “married,” and “divorced, widowed, and others.” Economic hardship was assessed based on self-reporting and dichotomized into two groups: “stable” and “unstable.” Domestic violence was described using the following two questions: “Are you currently experiencing physical or psychological abuse from your partner?” and “Were you physically or psychologically abused by your partner in the past?” with a dichotomous Yes/No response item.

### 2.3. Statistical Analysis

Multiple logistic regression analyses were performed to determine the effect of childhood maltreatment history upon PMS. After estimating a bivariate model, age was added to the covariates in Model 1. Moreover, marital status, living with a child or children, smoking, alcohol, economic hardship, and domestic violence were incorporated into Model 2. All analyses were conducted using Stata 14 software in 2019 (Stata Statistical Software: Release 14; StataCorp LLC, College Station, TX, USA).

## 3. Results

### 3.1. Summary of the Demographic Characteristics of The Study Participants

Table 1 summarizes the study participants’ demographic characteristics. The participants had a mean age of 35.1 (standard deviation (SD) 10.3) years. Approximately half of the women were never married (51.5%), and approximately a quarter were living with a child or children (26.8%). Furthermore, most women had never smoked (73.9%), were not taking alcohol (65.1%), did not experience economic hardship (56%), and did not experience domestic violence (90.0%). Those who experienced childhood maltreatment comprised 24.1%; they were more likely to be divorced or widowed, to have smoked at some point in their lives, to have experienced domestic violence, and to have suffered economic hardship than those who did not.

### 3.2. Bivariate and Multivariate Associations between Childhood Maltreatment History and PMS

Table 2 summarizes the bivariate and multivariate associations between childhood maltreatment history and PMS via multivariate-regression analysis using all data. In the crude model, those who experienced childhood maltreatment were 1.39 times more likely to have PMS (odds ratio (OR) = 1.39, 95% confidence interval (CI) = 1.15–1.68) than those who did not. Furthermore, even adjusting for age, those who experienced childhood maltreatment were more likely to have PMS (OR = 1.51, 95% CI = 1.25–1.84) than those who did not (Model 1). As shown in Model 2, there was slight OR attenuation (OR = 1.47, 95% CI = 1.20–1.81) when adjustments were made for age, marital status, living with a child or children, smoking, alcohol, economic hardship, and domestic violence.

### 3.3. Bivariate and Multivariate Associations between The Number of Types of Childhood Maltreatment and PMS

Table 3 summarizes the bivariate and multivariate associations between the number of types of childhood maltreatment and PMS. In the crude model, we observed that the OR associated with having PMS significantly increased for higher numbers of types of childhood maltreatment. The OR was 1.71 (95% CI = 1.20–2.44) for those who experienced two types of childhood maltreatment, whereas it was 1.62 (95% CI = 1.16–2.27) for those having experienced at least three types of child maltreatment. Notably, the association remained significant even after age adjustment.

### 3.4. Bivariate and Multivariate Associations between Each Type of Childhood Maltreatment History and PMS

Table 4 summarizes the bivariate and multivariate associations between each type of childhood maltreatment and PMS. Physical and emotional abuse were found to be significantly associated with PMS. As demonstrated in the crude model, those who experienced physical abuse were more likely to have PMS (OR = 1.95, 95% CI = 1.43–2.67) than those who did not. In Model 2, after adjusting for other covariates, those who experienced physical abuse remained more likely to have PMS (OR = 2.02, 95% CI = 1.47–2.79) than those who did not. Additionally, the adjusted model shows that those who experienced emotional abuse were also more likely to have PMS (OR = 1.62, 95% CI = 1.29–2.03) than those who did not. Experiences of emotional neglect, witnessing IPV, and sexual abuse showed no significant association with PMS.

## 4. Discussion

Women reporting histories of childhood maltreatment were observed to exhibit a significantly increased risk of PMS compared with those who did not. Particularly, childhood physical and emotional abuse had significant associations with PMS, whereas other types of childhood maltreatment (i.e., emotional neglect, a witness of IPV, or sexual abuse) did not.

Our findings were consistent with those of previous studies. The association between childhood abuse (emotional, physical, and sexual) and the onset of PMS has been examined in the past using the Nurses’ Health Study II, a prospective cohort study [14]. After adjustment for obesity, smoking, and other factors, women who had experienced emotional and physical abuse were found to be 1.6 times more likely to have PMS than those who had not. The same study showed that childhood sexual abuse was not associated with PMS, which also accords with our results. Therefore, we have shown that the association between childhood abuse and PMS in Americans can be extended to Japanese women. Although the Nurses’ Health Study involved only professional nurses aged 28 and up, the participants (women) in the present study were not necessarily professional nurses (i.e., they were drawn from the general population) and had ages ranging from 10 years old or higher. Thus, our findings suggest that the association between childhood maltreatment history and PMS can be extended to the general population and younger women.

The social structure of Japan has changed significantly in the last few decades, and the social burden on women has increased. Along with the fact that female-specific diseases such as PMS have increased, more women are aware of the existence of PMS in the information-oriented society, and more women are suffering from PMS. Because some previous studies have suggested that PMS may be a socially constructed disorder [3], the recent increase in recognition of PMS in Japan may be attributed to social construction. On the other hand, in Japan, child maltreatment has recently been socially recognized by the Child Guidance Center (which is similar to Child Protection Services in the United States or the United Kingdom), and reported cases were found to be 40 times more numerous than they were 20 years ago [22]. Such social–environmental change may have significantly contributed to the increase in the recognition of both child maltreatment and PMS. Given that both PMS and child maltreatment are susceptible to social construction, further studies should be conducted to examine the association between PMS and child maltreatment.

Our findings are expected to be useful in elucidating the etiology of PMS. Although several factors are intimately involved and complicated in the occurrence of PMS [23], the etiology of this syndrome remains unknown. One possible etiology lies in the serotonin pathway, that is, PMS symptoms can be effectively alleviated not only by oral administration of serotonin-selective reuptake inhibitors (SSRIs) but also by treatments that enhance serotonin [24]. Moreover, in patients suffering from serotonin-based nervous-system disorders, diets lacking in tryptophan (which is needed to synthesize serotonin) were reported to induce PMS [25]. Beyond this, premenstrual dysphoric disorder (PMDD) is associated with the short allele of a functional polymorphism in the serotonin-transporter gene (5-HTTLPR), leading to lower levels of serotonin [26]. Since the experience of childhood abuse can change the brain structure and function related to the serotonin system [27], this study may support the hypothesis that the serotonin pathway causes PMS.

Another possible pathway is brain structure. Fujisawa et al. reported that childhood abuse may shrink specific parts of the brain [28]; among those who experienced childhood abuse, the authors observed a decreased level of oxytocin. Considering the interplay between oxytocin and serotonin (that is, reduced oxytocin can induce lower levels of serotonin [29]), a history of childhood abuse may change the brain structure related to oxytocin levels and consequently change the serotonin levels, which may induce PMS.

## 5. Limitations

This study had several limitations. First, we used a self-reporting questionnaire to evaluate both childhood maltreatment history and PMS. Our results were therefore likely affected by common method bias. Our results were also subject to recall bias because the assessment of childhood maltreatment history was done retrospectively. To address this problem, future studies should make use of objective childhood maltreatment data from the Child Guidance Center. Second, it was not feasible to utilize the Japanese version of the Childhood Trauma Questionnaire [30] to assess childhood maltreatment history in the clinical setting; further studies should therefore be conducted using a validated detailed questionnaire. Third, sampling bias was unavoidable in this study since the participant pool was limited to two clinics in an urban area in Japan. Therefore, further population-based studies of this topic should be conducted. Fourth, this study employed a cross-sectional design, meaning that we cannot rule out reverse causation. Therefore, people with PMS may be more likely to remember (or overestimate) their childhood maltreatment history. However, we collected a relatively large sample size and considered multiple confounding factors; thus, the effect of reverse causation may be minimal or insufficient to impact our findings.

## 6. Conclusions

In this study, we observed that women reporting any childhood maltreatment history were 1.5 times more likely to experience PMS than those who did not. Particularly, childhood physical and emotional abuse showed stronger associations with PMS, whereas sexual abuse, emotional neglect, and witnessing IPV did not appear to be associated with PMS. In clinical settings for treatment of PMS, it may be helpful to assess patient’s childhood maltreatment histories. The findings of this study may suggest that addressing patients’ perceptions of childhood maltreatment using cognitive behavioral therapy might be an effective way to treat PMS, in addition to direct treatment of PMS symptoms. Thus, psychological therapy for childhood maltreatment may be a more effective way to address PMS than prescription drugs such as SSRI for PMS. Further intervention studies should be conducted to investigate the effectiveness of psychological therapy among patients with PMS and histories of childhood maltreatment.

## Figures and Tables

**Table 1:** Characteristics of respondents (*N* = 3815).

Characteristics of Respondents		Child Maltreatment History						*p*-Value
		Total (*N* = 3815)		None (*N* = 2894, 75.9%)		Any (*N* = 921, 24.1%)		
		*N* or Mean	% or SD	*N* or Mean	% or SD	*N* or Mean	% or SD	
Age		35.1	10.3	34.9	10.2	35.9	10.6	0.011
Age (category)	25 and older	3196	83.8	2432	84	764	83	0.438
	24 and younger	619	16.2	462	16	157	17	
Marital status	Never married	1966	51.5	1508	52.1	458	49.7	<0.001
	Married	1585	41.5	1226	42.4	359	39	
	Divorced, widowed, others	252	6.6	152	5.3	100	10.9	
Living with child/children	Yes	1021	26.8	769	26.6	252	27.4	0.832
	No	2791	73.2	2123	73.4	668	72.5	
Smoking	No	2818	73.9	2206	76.2	612	66.4	<0.001
	Used to smoke	573	15	403	13.9	170	18.5	
	Yes	362	9.5	242	8.4	120	13	
Alcohol	No	1332	34.9	973	33.6	359	39	0.005
	1–3 times per month	1293	33.9	1020	35.2	273	29.6	
	Once a week or more	1119	29.3	850	29.4	269	29.2	
Economic hardship	No	2136	56	1735	60	401	43.5	<0.001
	Yes	1638	42.9	1128	39	510	55.4	
Domestic violence	No	3408	89.3	2705	93.5	703	76.3	<0.001
	Yes	382	10	172	5.9	210	22.8	

**Table 2:** Bivariate and multivariate associations between childhood maltreatment history and premenstrual syndrome.

Predictors		Prevalence of PMS (%)	Bivariate		Model 1		Model 2	
			OR	95% CI	aOR	95% CI	aOR	95% CI
Any child maltreatment history	No	79.2	Ref		Ref		Ref	
	Yes	83.4	**1.39**	**1.15–1.68**	**1.51**	**1.25–1.84**	**1.47**	**1.20–1.81**
Age					**0.94**	**0.94–0.95**	**0.94**	**0.93–0.94**
Marital status	Never married	83.9					Ref	
	Married	78.2					**1.38**	**1.10–1.74**
	Divorced, widowed, others	63.5					**0.63**	**0.45–0.88**
Living with child/children	No	81.6					Ref	
	Yes	76.4					1.01	0.81–1.27
Smoking	No	79.7					Ref	
	Used to smoke	82.2					**1.41**	**1.12–1.80**
	Yes	84.3					1.29	0.95–1.75
Alcohol	No	78.9					Ref	
	1–3 times per month	83.8					**1.32**	**1.08–1.61**
	Once a week or more	80.7					1.22	0.99–1.49
Economic hardship	No	78.6					Ref	
	Yes	82.7					**1.32**	**1.11–1.57**
Domestic Violence	No	79.8					Ref	
	Yes	85.6					**1.57**	**1.15–2.15**

OR: odds ratios; bold: *p* < 0.05. Model 1: adjusted for age. Model 2: adjusted for age, marital status, living with child/children, smoking, alcohol, economic hardship, and domestic violence.

**Table 3:** Bivariate and multivariate associations between the number of types of childhood maltreatment and premenstrual syndrome.

Predictors		Crude		Model 1		Model 2	
		OR	95% CI	aOR	95% CI	aOR	95% CI
No. of child maltreatment history	0	Ref		Ref		Ref	
	1	1.13	0.87–1.46	1.19	0.91–1.55	1.32	0.93–1.63
	2	**1.71**	**1.20–2.44**	**1.87**	**1.30–2.69**	**1.88**	**1.28–2.75**
	3+	**1.62**	**1.16–2.27**	**1.82**	**1.29–2.57**	**1.60**	**1.11–2.29**
Age				**0.94**	**0.94–0.95**	**0.94**	**0.93–0.94**
Marital status	Never married					Ref	
	Married					**1.39**	**1.10–1.75**
	Divorced, Widowed, Others					**0.64**	**0.45–0.89**
Living with child/children	No					Ref	
	Yes					1.01	0.81–1.26
Smoking	No					Ref	
	Used to smoke					**1.41**	**1.10–1.80**
	Yes					1.28	0.94–1.74
Alcohol	No					Ref	
	1–3 times per month					**1.32**	**1.09–1.62**
	Once a week or more					**1.22**	**1.00–1.50**
Economic hardship	No					Ref	
	Yes					**1.32**	**1.11–1.56**
Domestic Violence	No					Ref	
	Yes					**1.53**	**1.12–2.10**

OR: odds ratios; bold: *p* < 0.05. Model 1: age adjustment. Model 2: age adjustment, marital status, living with child/children, smoking, alcohol, economic hardship, and domestic violence.

**Table 4:** Bivariate and multivariate associations between each type of childhood maltreatment history and premenstrual syndrome.

Each Type of Childhood Maltreatment History	Crude		Model 1		Model 2	
	OR	95% CI	aOR	95% CI	aOR	95% CI
Emotional neglect	1.16	0.90–1.51	**1.32**	**1.01–1.73**	1.20	0.90–1.59
Witnessing intimate-partner violence	1.21	0.91–1.62	**1.40**	**1.04–1.89**	1.26	0.92–1.73
Physical abuse	**1.95**	**1.43–2.67**	**2.02**	**1.47–2.79**	**1.89**	**1.35–2.64**
Emotional abuse	**1.53**	**1.23–1.92**	**1.62**	**1.29–2.03**	**1.55**	**1.21–1.98**
Sexual abuse	1.60	0.95–2.69	**1.76**	**1.03–3.01**	1.50	0.87–2.58

OR: odds ratios; bold: *p* < 0.05.

## Data Availability

Due to the nature of this research, participants of this study did not agree for their data to be shared publicly, so supporting data is not available.

## References

[1] Heinemann L.A., Minh T.D., Filonenko A., Uhl-Hochgraber K. (2010). Explorative evaluation of the impact of severe premenstrual disorders on work absenteeism and productivity. Womens Health Issues.

[2] Yonkers K.A., O’Brien P.M., Eriksson E. (2008). Premenstrual syndrome. Lancet.

[3] Ussher J.M., Perz J. (2013). PMS as a process of negotiation: Women’s experience and management of premenstrual distress. Psychol. Health.

[4] Tanaka E., Momoeda M., Osuga Y., Rossi B., Nomoto K., Hayakawa M., Kokubo K., Wang E.C. (2013). Burden of menstrual symptoms in Japanese women—An analysis of medical care-seeking behavior from a survey-based study. Int. J. Womens Health.

[5] Takeda T., Tasaka K., Sakata M., Murata Y. (2006). Prevalence of premenstrual syndrome and premenstrual dysphoric disorder in Japanese women. Arch. Womens Ment. Health.

[6] Angst J., Sellaro R., Merikangas K.R., Endicott J. (2001). The epidemiology of perimenstrual psychological symptoms. Acta Psychiatr. Scand..

[7] Rapkin A.J., Winer S.A. (2009). Premenstrual syndrome and premenstrual dysphoric disorder: Quality of life and burden of illness. Expert Rev. Pharmacoecon. Outcomes Res..

[8] Borenstein J.E., Dean B.B., Endicott J., Wong J., Brown C., Dickerson V., Yonkers K.A. (2003). Health and economic impact of the premenstrual syndrome. J. Reprod. Med..

[9] Grady-Weliky T.A. (2003). Clinical practice. Premenstrual dysphoric disorder. N. Engl. J. Med..

[10] Futterman L.A., Rapkin A.J. (2006). Diagnosis of premenstrual disorders. J. Reprod. Med..

[11] Felitti V.J., Anda R.F., Nordenberg D., Williamson D.F., Spitz A.M., Edwards V., Koss M.P., Marks J.S. (1998). Relationship of childhood abuse and household dysfunction to many of the leading causes of death in adults. The Adverse Childhood Experiences (ACE) Study. Am. J. Prev. Med..

[12] Fujiwara T., Kawakami N., World Mental Health Japan Survey (2011). Association of childhood adversities with the first onset of mental disorders in Japan: Results from the World Mental Health Japan, 2002–2004. J. Psychiatr. Res..

[13] Koci A., Strickland O. (2007). Relationship of adolescent physical and sexual abuse to perimenstrual symptoms (PMS) in adulthood. Issues Ment. Health Nurs..

[14] Bertone-Johnson E.R., Whitcomb B.W., Missmer S.A., Manson J.E., Hankinson S.E., Rich-Edwards J.W. (2014). Early life emotional, physical, and sexual abuse and the development of premenstrual syndrome: A longitudinal study. J. Womens Health.

[15] Organisation for Economic Co-Operation and Development OECD Family Database. http://www.oecd.org/els/soc/SF3_4_Family_violence_Jan2013.pdf.

[16] The Japanese Society of Obstetrics and Gynecology Obstetrics (1999). Obstetrics and Gynecology Glossary.

[17] AOCG Practice Bulletin (2001). Premenstrual syndrome. Int. J. Gyaecol. Obstet..

[18] Isumi A., Fujiwara T. (2016). Association of adverse childhood experiences with shaking and smothering behaviors among Japanese caregivers. Child Abuse Negl..

[19] Doi S., Fujiwara T. (2019). Combined effect of adverse childhood experiences and young age on self-harm ideation among postpartum women in Japan. J. Affect. Disord..

[20] Amemiya A., Fujiwara T., Murayama H., Tani Y., Kondo K. (2018). Adverse Childhood Experiences and Higher-Level Functional Limitations among Older Japanese People: Results from the JAGES Study. J. Gerontol. A Biol. Sci. Med. Sci..

[21] Matsuyama Y., Fujiwara T., Aida J., Watt R.G., Kondo N., Yamamoto T., Kondo K., Osaka K. (2016). Experience of childhood abuse and later number of remaining teeth in older Japanese: A life-course study from Japan Gerontological Evaluation Study project. Community Dent. Oral Epidemiol..

[22] Tanaka R. (2011). Child abuse as a social problem. J. Educ. Sociol..

[23] Halbreich U. (2003). The etiology, biology, and evolving pathology of premenstrual syndromes. Psychoneuroendocrinology.

[24] Landen M., Eriksson O., Sundblad C., Andersch B., Naessen T., Eriksson E. (2001). Compounds with affinity for serotonergic receptors in the treatment of premenstrual dysphoria: A comparison of buspirone, nefazodone and placebo. Psychopharmacology.

[25] Menkes D.B., Coates D.C., Fawcett J.P. (1994). Acute tryptophan depletion aggravates premenstrual syndrome. J. Affect. Disord..

[26] Gingnell M., Comasco E., Oreland L., Fredrikson M., Sundstrom-Poromaa I. (2010). Neuroticism-related personality traits are related to symptom severity in patients with premenstrual dysphoric disorder and to the serotonin transporter gene-linked polymorphism 5-HTTPLPR. Arch. Womens Ment. Health.

[27] Miller J.M., Kinnally E.L., Ogden R.T., Oquendo M.A., Mann J.J., Parsey R.V. (2009). Reported childhood abuse is associated with low serotonin transporter binding in vivo in major depressive disorder. Synapse.

[28] Fujisawa T.X., Nishitani S., Takiguchi S., Shimada K., Smith A.K., Tomoda A. (2019). Oxytocin receptor DNA methylation and alterations of brain volumes in maltreated children. Neuropsychopharmacology.

[29] Lefevre A., Richard N., Jazayeri M., Beuriat P.A., Fieux S., Zimmer L., Duhamel J.R., Sirigu A. (2017). Oxytocin and Serotonin Brain Mechanisms in the Nonhuman Primate. J. Neurosci..

[30] Mizuki R., Fujiwara T. (2020). Validation of the Japanese version of the Childhood Trauma Questionnaire-Short Form (CTQ-J). Psychol. Trauma.

